# Utility of the detection of *Plasmodium *parasites for the diagnosis of malaria in endemic areas

**DOI:** 10.1186/1471-2334-6-81

**Published:** 2006-05-02

**Authors:** Thomas V Perneger, Thomas Szeless, André Rougemont

**Affiliations:** 1 Institute of Social and Preventive Medicine, University of Geneva, CH-1211 Geneva, Switzerland; 2 Quality of Care Service, University Hospitals of Geneva, CH-1211 Geneva, Switzerland

## Abstract

**Background:**

In populations where the prevalence of infection with *Plasmodium *parasites is high, blood tests that identify *Plasmodium *parasites in patients with fever may lead to false positive diagnosis of malaria-disease. We characterised the diminishing value of the parasite detection test as a function of the prevalence of infection.

**Methods:**

We computed the ability of the parasite detection test to identify malaria at various levels of prevalence (0% to 90%), assuming plausible estimates of sensitivity (95% and 85%) and specificity (99% and 95%) for the detection of parasites. In each situation, we computed likelihood ratios of malaria (or absence of malaria) for positive and negative parasite detection tests. Likelihood ratios were classified as clinically useful (≥ 10), intermediate (5–10), or unhelpful (<5).

**Results:**

Likelihood ratios of positive tests were strongly related to the prevalence of infection in the general population: a positive test was unhelpful when the prevalence was 20% or more, and useful only when prevalence was 5% or less. The sensitivity and specificity of the test had little influence on these results. Likelihood ratios of negative tests were clinically useful when prevalence was 70% or less, but only for high levels of sensitivity (95%). If sensitivity was low (85%), the negative test was at best of intermediate utility, and was unhelpful if the prevalence of asymptomatic infection exceeded 30%.

**Conclusion:**

Identification of *Plasmodium *parasites supports a diagnosis of malaria only in areas where the prevalence of *Plasmodium *infection is low. Wherever this prevalence exceeds about 20%, a positive test is clinically unhelpful.

## Background

In current practice, the diagnosis of malaria in a patient in whom this disease is suspected rests on the identification of *Plasmodium* parasites in the patient's blood [1]. This diagnostic method is currently recommended by the World Health Organization [2]. Parasite detection tests include the classical methods of the thick or thin blood smear, and various rapid diagnostic tests [3].

This approach works well in cases of traveller's malaria, and more generally whenever the patient is unlikely to be an asymptomatic carrier of *Plasmodium* parasites. However, in populations where malaria is common, many people are infected by Plasmodium parasites without being sick. In such situations, using the presence of parasites as a criterion for diagnosing malaria in a febrile patient will lead to over-diagnosis of malaria, unnecessary anti-malarial treatments, and missed diagnoses of other febrile illnesses. While this point has been made by experts previously [1], it is usually framed in general terms, without precise guidance as to when a positive or a negative parasite detection test is clinically useful, and when it is not. In this paper, we examine the influence of the prevalence of asymptomatic parasitic infection on the clinical utility of parasite detection for the diagnosis of malaria in patients with fever.

## Methods

We examined various hypotheses regarding test performance and the prevalence of parasitic infection in the general population. A prevalence <20% is sometimes qualified as low, 20–50% as intermediate, and >50% as high.

We distinguish between the ability of a test to detect parasites in a person's blood, i.e., infection, from its ability to identify malaria as clinical disease in a patient with fever. The former is a general characteristic of the test, independent of the prevalence of parasitic infection. The latter pertains to a test used in specific epidemiologic circumstances, and is related to the prevalence of infection (Box 1).

### Assessing the utility of a diagnostic test

Classic measures of the performance of a diagnostic test are its sensitivity (Sn) – i.e., the proportion of positive tests among those with the disease – and its specificity (Sp) – i.e., the proportion of negative tests among those without the disease. However, these statistics are not directly applicable in clinical practice, for if we knew who had the disease and who did not, we would not need the test. The clinician is typically interested in the test's positive predictive value – i.e., the proportion of diseased people among those with a positive test, and in its negative predictive value – i.e., the proportion of non-diseased people among those with a negative test. The drawback of these statistics is that they depend strongly on the pre-test probability of disease, which varies from patient to patient.

The statistic that best characterises the clinical utility of a test result is the likelihood ratio (LR) [5-7]. The likelihood ratio is the ratio of probabilities of a given test result under the 2 hypotheses under consideration – i.e., presence versus absence of disease. The likelihood ratio of a positive test is given by LR+ = Sn/(1 - Sp), and the likelihood ratio of a negative test, by LR- = (1 - Sn)/Sp. To facilitate comparisons [6,7], likelihood ratios of a negative test are sometimes shown for the *absence *of disease, i.e., LR-_0 _= Sp/(1 - Sn). This merely inverses the likelihood ratio: a likelihood ratio of 0.1 for the disease becomes 10 for the absence of disease. We have adopted this convention in this paper.

**Figure 1:**
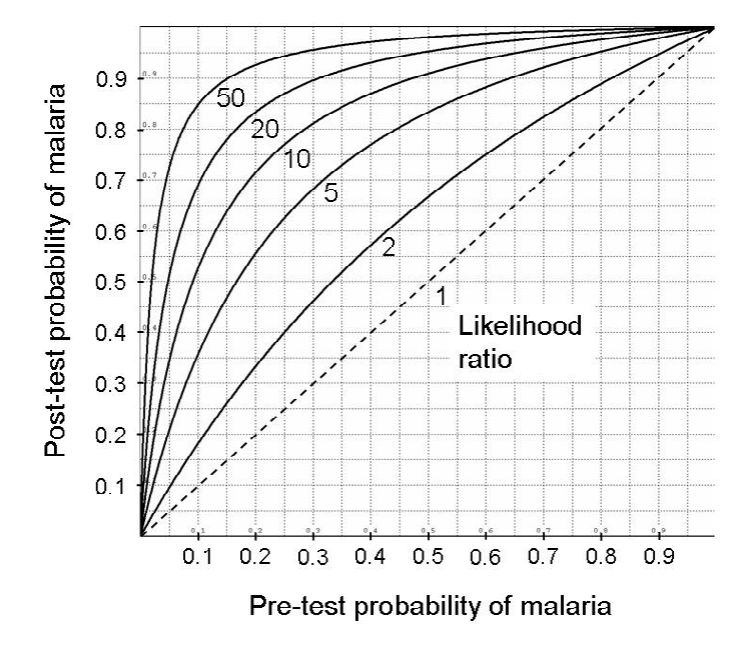
Post-test probability of malaria, as a function of pre-test probability of malaria, for 5 levels of the likelihood ratio of a positive test (2, 5, 10, 20 or 50). An uninformative test (likelihood ratio = 1) is shown as a diagonal dotted line.

The likelihood ratio allows the computation of the post-test probability of disease. According to the Bayes' theorem, post-test odds of disease are the product of the likelihood ratio and the pre-test odds [5-7]. Because probabilities can be converted into odds (O = P/(1 - P)), and odds into probabilities (P = O/(O + 1)), the likelihood ratio allows the transformation of a pre-test probability into a post-test probability (Figure 1). For example, if the pre-test probability of disease was 20% (i.e., pre-test odds = 1/4), and the test was positive, with a likelihood ratio of 8, the post-test odds of disease would be 2 (i.e., 8 × (1/4)), and the corresponding probability 66.7% (i.e., 2/(2+1)).

This is how likelihood ratios can be interpreted [6]: a likelihood ratio is clinically useful if it equals 10 or more, of intermediate usefulness between 5 and 10, and clinically unhelpful below 5.

### Detection of parasites in blood

To examine the performance of a parasite detection test for determining the presence or absence of *Plasmodium *parasites in the blood, we assumed a sensitivity of either 85% or 95%, and a specificity of either 95% or 99%. This means that among 100 persons who harbour parasites, the test will be positive for 85 or 95. The remaining are "false negatives", due to technical problems, or to the parasite being absent from peripheral blood for a portion of its life cycle [4]. Similarly, among 100 persons who do not harbour parasites, the test will be falsely positive for 1 to 5, due to various laboratory errors. These values of sensitivity and specificity can be considered as realistic in a well equipped and expertly managed laboratory.

### Diagnosis of malaria

In a clinical context, the purpose of the test is not merely to detect parasites in the blood, but to establish or rule out the diagnosis of malaria in patients in whom the disease is suspected. Three types of patients should be considered (Table 1): a) patients with malaria, all of whom can be assumed to harbour parasites, b) patients with fever from another origin who are also infected – but not ill – with *Plasmodium*, and c) patients with fever from another origin who are not infected with *Plasmodium*. The probability that patients with fever from another origin are also infected with *Plasmodium *equals roughly the prevalence of parasitic infection in the general population. If this prevalence is nil, as is the case in much of the Western world, the performance of the test for diagnosing malaria is the same as for identifying the presence of parasites.

**Table 1:** Performance of *Plasmodium* detection tests in endemic populations: a) detection of the presence of parasites, b) diagnosis of a malaria. The parameters considered include the specificity of the test for the detection of parasites in blood (Sn_p_), the corresponding specificity (Sp_p_), the prevalence of malaria infection in the general population (x), and the pre-test probability of a malaria attack in a patient with fever (m)

**a) Detection of parasites**			
	Disease status		
	
	Malaria	Other febrile illness, parasites present	Other febrile illness, parasites absent
	
Positive parasite detection test	Sn_p_	Sn_p_	1-Sp_p_
Negative parasite detection test	1-Sn_p_	1-Sn_p_	Sp_p_
Column totals	1	1	1
Prevalence of infection in the general population	-	x	1-x

**b) Detection of malaria (disease)**			

	Malaria	Other febrile illness	
	
Positive parasite detection test	Sn_p_	Sn_p_·x + (1-Sp_p_)·(1-x)	
Negative parasite detection test	1-Sn_p_	(1-Sn_p_)·x + Sp_p_·(1-x)	
Column totals	1	1	
Pre-test probability of malaria	m	1-m	

The sensitivity of the parasite detection test for detecting malaria (Sn_m_) will be the same as its sensitivity for detecting parasites (Sn_p_). However, the specificity of the test for detecting malaria (Sp_m_) will be influenced by the prevalence of infection, because the population under consideration is a mixture of two subgroups: patients who are infected with *Plasmodium *(proportion x), and of those who are not (proportion 1-x). The specificity of the test for detecting malaria becomes Sp_m _= Sp_p_·(1 - x) + (1 - Sn_p_)·x, and its complement, 1 - Sp_m _= (1 - Sp_p_)·(1 - x) + Sn_p_·x (Box 1).

Consequently, the likelihood ratio of a positive test for the presence of malaria disease becomes LR+m=Snp(1−Spp)⋅(1−x)+Snp⋅x
 MathType@MTEF@5@5@+=feaafiart1ev1aaatCvAUfKttLearuWrP9MDH5MBPbIqV92AaeXatLxBI9gBaebbnrfifHhDYfgasaacH8akY=wiFfYdH8Gipec8Eeeu0xXdbba9frFj0=OqFfea0dXdd9vqai=hGuQ8kuc9pgc9s8qqaq=dirpe0xb9q8qiLsFr0=vr0=vr0dc8meaabaqaciaacaGaaeqabaqabeGadaaakeaacqqGmbatcqqGsbGucqGHRaWkdaWgaaWcbaGaeeyBa0gabeaakiabg2da9maalaaabaGaee4uamLaeeOBa42aaSbaaSqaaiabbchaWbqabaaakeaacqGGOaakcqaIXaqmcqGHsislcqqGtbWucqqGWbaCdaWgaaWcbaGaeeiCaahabeaakiabcMcaPiabgwSixlabcIcaOiabigdaXiabgkHiTiabbIha4jabcMcaPiabgUcaRiabbofatjabb6gaUnaaBaaaleaacqqGWbaCaeqaaOGaeyyXICTaeeiEaGhaaaaa@4E92@. The likelihood ratio of a negative test for the *absence *of malaria is LR−0m=Spp⋅(1−x)+(1−Snp)⋅x1−Snp
 MathType@MTEF@5@5@+=feaafiart1ev1aaatCvAUfKttLearuWrP9MDH5MBPbIqV92AaeXatLxBI9gBaebbnrfifHhDYfgasaacH8akY=wiFfYdH8Gipec8Eeeu0xXdbba9frFj0=OqFfea0dXdd9vqai=hGuQ8kuc9pgc9s8qqaq=dirpe0xb9q8qiLsFr0=vr0=vr0dc8meaabaqaciaacaGaaeqabaqabeGadaaakeaacqqGmbatcqqGsbGucqGHsisldaWgaaWcbaGaeeimaaJaeeyBa0gabeaakiabg2da9maalaaabaGaee4uamLaeeiCaa3aaSbaaSqaaiabbchaWbqabaGccqGHflY1cqGGOaakcqaIXaqmcqGHsislcqqG4baEcqGGPaqkcqGHRaWkcqGGOaakcqaIXaqmcqGHsislcqqGtbWucqqGUbGBdaWgaaWcbaGaeeiCaahabeaakiabcMcaPiabgwSixlabbIha4bqaaiabigdaXiabgkHiTiabbofatjabb6gaUnaaBaaaleaacqqGWbaCaeqaaaaaaaa@5157@.

Note that the proportion of *Plasmodium* carriage in the population (denoted by x in Table 1) is distinct from the pre-test probability of malaria in a given patient with fever (denoted by m in Table 1). The former is a characteristic of the population to which the patient belongs. The latter depends on various patient-related factors, such as the intensity and temporal pattern of fever, presence or absence of an enlarged spleen, anemia, pregnancy, age, exposure to mosquito bites, season, likelihood of alternate diseases, etc.

### Effect of a higher threshold for a positive test

Thus far we have assumed that the test would perform equally well in patients who suffer from malaria and in those who are infected but not ill with malaria. But because parasite levels are likely higher in the former group, the test may discriminate better if a higher parasite threshold is used to diagnose malaria – say, greater than 1000 parasites per μL instead of greater than 0. Raising the diagnostic threshold will decrease the sensitivity of the test, but less so in patients with malaria (Sn_p-malaria_) than in other patients (Sn_p-other_). At the same time, raising the threshold will increase the specificity of the test to virtually 100%. Under these conditions, the likelihood ratio of a positive test for the presence of malaria becomes: LR+m=1x⋅Snp−malariaSnp−other
 MathType@MTEF@5@5@+=feaafiart1ev1aaatCvAUfKttLearuWrP9MDH5MBPbIqV92AaeXatLxBI9gBaebbnrfifHhDYfgasaacH8akY=wiFfYdH8Gipec8Eeeu0xXdbba9frFj0=OqFfea0dXdd9vqai=hGuQ8kuc9pgc9s8qqaq=dirpe0xb9q8qiLsFr0=vr0=vr0dc8meaabaqaciaacaGaaeqabaqabeGadaaakeaacqqGmbatcqqGsbGucqGHRaWkdaWgaaWcbaGaeeyBa0gabeaakiabg2da9maalaaabaGaeGymaedabaGaeeiEaGhaaiabgwSixpaalaaabaGaee4uamLaeeOBa42aaSbaaSqaaiabbchaWjabgkHiTiabb2gaTjabbggaHjabbYgaSjabbggaHjabbkhaYjabbMgaPjabbggaHbqabaaakeaacqqGtbWucqqGUbGBdaWgaaWcbaGaeeiCaaNaeyOeI0Iaee4Ba8MaeeiDaqNaeeiAaGMaeeyzauMaeeOCaihabeaaaaaaaa@51B6@, and the likelihood ratio of a negative test for the absence of malaria becomes LR−0m=1−x⋅Snp−other1−Snp−malaria
 MathType@MTEF@5@5@+=feaafiart1ev1aaatCvAUfKttLearuWrP9MDH5MBPbIqV92AaeXatLxBI9gBaebbnrfifHhDYfgasaacH8akY=wiFfYdH8Gipec8Eeeu0xXdbba9frFj0=OqFfea0dXdd9vqai=hGuQ8kuc9pgc9s8qqaq=dirpe0xb9q8qiLsFr0=vr0=vr0dc8meaabaqaciaacaGaaeqabaqabeGadaaakeaacqqGmbatcqqGsbGucqGHsisldaWgaaWcbaGaeeimaaJaeeyBa0gabeaakiabg2da9maalaaabaGaeGymaeJaeyOeI0IaeeiEaGNaeyyXICTaee4uamLaeeOBa42aaSbaaSqaaiabbchaWjabgkHiTiabb+gaVjabbsha0jabbIgaOjabbwgaLjabbkhaYbqabaaakeaacqaIXaqmcqGHsislcqqGtbWucqqGUbGBdaWgaaWcbaGaeeiCaaNaeyOeI0IaeeyBa0MaeeyyaeMaeeiBaWMaeeyyaeMaeeOCaiNaeeyAaKMaeeyyaegabeaaaaaaaa@5562@.

### Analysis of test performance

We considered a range of prevalence of infection in the general population between 0% and 90%, and plausible values of sensitivity (0.85 and 0.95) and specificity (0.95 and 0.99) of the test for the detection of parasites. We show likelihood ratios of malaria for a positive test (Table 2), and of absence of malaria for a negative test (Table 3). We also explored the effect of raising the diagnostic threshold, assuming that this will lower the sensitivity of the test, but less so in patients with malaria than in patients with an asymptomatic infection (Table 4). The computations were done using macro functions written for SPSS software.

**Table 2:** Likelihood ratios of malaria when the parasite detection test is positive, according to the prevalence of infection in the general population, and to the sensitivity and specificity of the test for the detection of parasites. Clinically useful tests are in bolded type, tests of intermediate utility are in standard type, clinically unhelpful tests in italics

Prevalence of infection in the general population	Sensitivity = 0.95		Sensitivity = 0.85	
	
	Spec. = 0.99	Spec. = 0.95	Spec. = 0.99	Spec. = 0.95
0%	**95.0**	**19.0**	**85.0**	**17.0**
0.1%	**86.8**	**18.7**	**78.4**	**16.7**
1%	**49.0**	**16.1**	**46.2**	**14.7**
2%	**33.0**	**14.0**	**31.7**	**12.9**
5%	**16.7**	**10.0**	**16.4**	9.4
10%	9.1	6.8	9.0	6.5
15%	6.3	5.1	6.2	5.0
20%	*4.8*	*4.1*	*4.8*	*4.0*
30%	*3.2*	*3.0*	*3.2*	*3.0*
40%	*2.5*	*2.3*	*2.5*	*2.3*
50%	*2.0*	*1.9*	*2.0*	*1.9*
60%	*1.7*	*1.6*	*1.6*	*1.6*
70%	*1.4*	*1.4*	*1.4*	*1.4*
80%	*1.2*	*1.2*	*1.2*	*1.2*
90%	*1.1*	*1.1*	*1.1*	*1.1*

**Table 3:** Likelihood ratios of the *absence *of malaria when the parasite detection test is negative, according to the prevalence of infection in the general population, and to the sensitivity and specificity of the test for the detection of parasites. Clinically useful tests are in bolded type, tests of intermediate utility are in standard type, clinically unhelpful tests in italics

Prevalence of infection in the general population	Sensitivity = 0.95		Sensitivity = 0.85	
	
	Spec. = 0.99	Spec. = 0.95	Spec. = 0.99	Spec. = 0.95
0%	**19.8**	**19.0**	6.6	6.3
0.1%	**19.8**	**19.0**	6.6	6.3
1%	**19.6**	**18.8**	6.5	6.2
2%	**19.4**	**18.6**	6.5	6.2
5%	**18.9**	**18.1**	6.3	6.1
10%	**17.9**	**17.2**	6.0	5.8
15%	**17.0**	**16.3**	5.8	5.5
20%	**16.0**	**15.4**	5.5	5.3
30%	**14.2**	**13.6**	*4.9*	*4.7*
40%	**12.3**	**11.8**	*4.7*	*4.2*
50%	**10.4**	**10.0**	*3.8*	*3.7*
60%	8.5	8.2	*3.2*	*3.1*
70%	6.6	6.4	*2.7*	*2.6*
80%	*4.8*	*4.6*	*2.1*	*2.1*
90%	*2.9*	*2.8*	*1.6*	*1.5*

**Table 4:** Likelihood ratios of positive test for presence of malaria if a high threshold is used to define a positive test. The sensitivity of the test is reduced, but less so for patients with malaria than for other infected individuals, and specificity is considered to be perfect (100%). Clinically useful tests are in bolded type, tests of intermediate utility are in standard type, clinically unhelpful tests in italics

Prevalence of infection in the general population	Sensitivity of test in patients with malaria: 0.80. Sensitivity of test in other infected patients:	
	
	0.60	0.40
0%	**infinite**	**infinite**
0.1%	**1333**	**2000**
1%	**133**	**200**
2%	**67.7**	**100**
5%	**26.7**	**40.0**
10%	**13.3**	**20.0**
15%	8.9	**13.3**
20%	6.7	**10**
30%	*4.4*	6.7
40%	*3.3*	*5.0*
50%	*2.7*	*4.0*
60%	*2.2*	*3.3*
70%	*1.9*	*2.9*
80%	*1.7*	*2.5*
90%	*1.5*	*2.2*

## Results

### Detection of parasites

The likelihood ratios for the detection of parasites correspond to those of the detection of malaria when the prevalence of asymptomatic infection is assumed to be nil (first result lines in Tables 2 and 3). For a positive test, the likelihood ratio was very high (95 and 85) when specificity was modelled as 0.99, and is still clinically useful (at 19 and 17) when specificity was assumed to be 0.95 (Table 2). In contrast, the likelihood ratio of a negative test depended on the value of sensitivity: it was close to 20 when sensitivity was modelled as 0.95, but of only borderline utility, around 6, when sensitivity was entered as 0.85 (Table 3).

### Detection of malaria

Likelihood ratios of malaria for a positive test varied considerably with the prevalence of *Plasmodium* infection (Table 2). When the prevalence was assumed to be 5% or less, the likelihood ratios were about 10 or more, in the clinically useful range. On the other hand, for a prevalence of 20% or more, the likelihood ratio dropped below 5, which would not be considered clinically useful. At low values of prevalence, the specificity of the test influenced considerably test performance. Whether sensitivity was modelled as 0.85 or as 0.95 was of little import for the likelihood ratios.

The impact of the prevalence of infection on the usefulness of a negative test was also notable (Table 3). The negative test remained clinically useful up to a prevalence of about 50%, as long as the sensitivity was high (0.95). However, even a negative test was not clinically useful when the prevalence exceeded 70%, or if the sensitivity of the test was assumed to be 0.85.

### Impact of a higher threshold for a positive test

Likelihood ratios of presence of malaria for a positive test improved when the diagnostic threshold was increased. If the change of threshold decreased sensitivity to 0.80 in patients with malaria and to 0.60 in patients with asymptomatic infection, a positive test was clinically useful up to a prevalence of infection of 10% (Table 4). If the sensitivities were 0.80 and 0.40, the test became useful up to a prevalence of 20%. In contrast, such a change of threshold virtually abolished the utility of a negative test. Even in the best case scenario (prevalence of 0%), the likelihood ratio of a negative test for absence of malaria was 5, and it was less than 5 in all other situations (detailed results not shown).

## Discussion

Globally, this analysis confirms that a positive parasite detection test is unhelpful in establishing the diagnosis of malaria in highly endemic areas [1], and defines a range of situations where a negative test is useful in excluding the diagnosis. Even in areas of intermediate prevalence of infection in the general population (20%–50%), a positive test for *Plasmodium* parasites in the blood does not establish the diagnosis of malaria. In such situations, the likelihood ratio is 5 or less, which will push the post-test probability of malaria above 90% only if the pre-test probability was already very high, greater than 70%. In highly endemic areas (>50%), a positive test is virtually meaningless, with likelihood ratios <2. While the general direction of these results is unsurprising, our study provides for the first time a definition of circumstances in which a parasite detection test is or is not useful for the diagnosis of malaria.

### Positive parasite detection test

The likelihood ratio of a positive test depends strongly on the prevalence of infection in the general population, and is hardly influenced by the sensitivity or specificity of the test, within the range we considered. In fact, the formula for the likelihood ratio of a positive test (LR(+)_m_) implies that this ratio cannot exceed the inverse of the prevalence of infection (or 1/x), even if both sensitivity and specificity *for the detection of parasites *are a perfect 100%. For example, if half of the population is infected by *Plasmodium* parasites, the likelihood ratio cannot exceed 2. Consequently, if prevalence of infection is high, ruling in malaria cannot be improved much by the development of more sensitive parasite detection tests, such as those based on the polymerase chain reaction assays [9]. Therefore a positive *Plasmodium* detection test has little utility in populations where malaria is most frequent, as in Africa, where most of the burden of mortality and morbidity of malaria occurs [10].

Using a higher parasite density threshold to consider a test as positive may improve the value of a positive test [11]. This is because raising the threshold will reduce the sensitivity among patients who harbour parasites but do not have clinical malaria more than among patients with malaria. However, even this strategy will not extend the usefulness of the positive test beyond a prevalence of asymptomatic infection greater than 20%. Furthermore, this strategy will invalidate the utility of a negative parasite detection test.

### Negative parasite detection test

In contrast, the diagnostic contribution of the *negative *test varied considerably with the sensitivity of the test. When sensitivity was deemed equal to 0.95, the negative test produced high, clinically useful likelihood ratios up to a prevalence of infection of 70%, allowing the exclusion of malaria even in populations where *Plasmodium* infection is highly prevalent. On the other hand, when sensitivity was assumed to be only 0.85, the likelihood ratios of a negative test were lower, and exceeded 5 only up to a prevalence of asymptomatic infection of about 20%. Therefore the sensitivity of the *Plasmodium* detection test is critical for ruling out malaria in highly endemic populations. Ruling out malaria in patients with fever is important, to avoid unnecessary treatment with antimalarial drugs, which is common in highly endemic countries [1], and to provide appropriate etiologic treatment to the patients [12]. In this paper, we do not address the issue of malaria control at the population level, which may or may not include the treatment of asymptomatic carriers.

Good quality laboratory tests to rule out *Plasmodium* infection are therefore important, especially in highly endemic areas. Our estimates assumed rather high values of sensitivity and specificity of the parasite detection test. In precarious field conditions, these test properties may be worse [13,14]. Lower sensitivity would considerably reduce the clinical usefulness of a negative test. Unfortunately, given the insufficient quality of many field laboratories, the practical contribution of a *Plasmodium* detection test to the diagnosis of malaria is often limited [15].

### Improving the diagnosis of malaria

Establishing the diagnosis of clinical malaria in endemic areas remains a challenge. As mentioned before, detection of *Plasmodium* infection in a patient presenting with a febrile acute illness is not a sufficient basis for the diagnosis. A useful contribution would be the identification of parameters, whether clinical variables or laboratory measurements, that distinguish patients who suffer from malaria from patients who have another febrile illness and merely harbour *Plasmodium* parasites. If clinical algorithms that yield a high enough pre-test probability of malaria were available, even a positive *Plasmodium* detection test associated with a modest likelihood ratio would help establish the diagnosis.

Several clinical algorithms have been proposed [16-20], but none seems sufficiently accurate as a basis for the diagnosis. A key difficulty in developing good diagnostic algorithms is the lack of a gold standard for diagnosing malaria. One cannot aim at a target that remains invisible. Development of adequate technologies to diagnose malaria in endemic areas remains an important and difficult public health challenge.

The difficulty in establishing the diagnosis of malaria increases with the prevalence of infection. It is noteworthy that reliance on positive *Plasmodium* detection tests remains justified in patients whose probability of asymptomatic parasitic infection is low, such as among travellers [14]. More generally, the issue of malaria diagnosis explored in this paper illustrates the difficulty in transferring a technological solution that is well suited to the developed world to other contexts.

## Conclusion

In populations where the prevalence of *Plasmodium* infection is intermediate or high, a positive *Plasmodium* detection test does not help establish the diagnosis of malaria.

## Abbreviations

Sn: Sensitivity

Sp: Specificity

LR: Likelihood ratio

## Competing interests

The author(s) declare that they have no competing interests.

## Authors' contributions

TVP and AR proposed the study and the study design. All authors discussed the interpretation of the results. TVP developed the statistical models, analysed data, and wrote the first draft. AR and TS performed the literature review and revised the paper.

## Pre-publication history

The pre-publication history for this paper can be accessed here:


